# S-Adenosylhomocysteine Is a Useful Metabolic Factor in the Early Prediction of Septic Disease Progression and Death in Critically Ill Patients: A Prospective Cohort Study

**DOI:** 10.3390/ijms241612600

**Published:** 2023-08-09

**Authors:** Franz-Simon Centner, Jochen J. Schoettler, Kathrin Brohm, Sonani Mindt, Evelyn Jäger, Bianka Hahn, Tanja Fuderer, Holger A. Lindner, Verena Schneider-Lindner, Joerg Krebs, Michael Neumaier, Manfred Thiel

**Affiliations:** 1 Department of Anesthesiology, Surgical Intensive Care Medicine and Pain Medicine, Medical Faculty Mannheim, University Medical Center Mannheim, University of Heidelberg, Theodor-Kutzer-Ufer 1-3, 68167 Mannheim, Germany; franz-simon.centner@umm.de (F.-S.C.); jochen.schoettler@umm.de (J.J.S.); kathrin.brohm@merckgroup.com (K.B.); bianka.hahn@medma.uni-heidelberg.de (B.H.); tanja.fuderer@medma.uni-heidelberg.de (T.F.); holgera.lindner@medma.uni-heidelberg.de (H.A.L.); verena.schneider-lindner@medma.uni-heidelberg.de (V.S.-L.); joerg.krebs@umm.de (J.K.); 2 Merck KGaA (SQ-Animal Affairs), Frankfurterstrasse 250, 64293 Darmstadt, Germany; 3 Institute for Clinical Chemistry, Medical Faculty Mannheim, University Medical Center Mannheim, University of Heidelberg, Theodor-Kutzer-Ufer 1-3, 68167 Mannheim, Germany; sonani.mindt@klinikum-passau.de (S.M.); jaeger.evelyn@umm.de (E.J.); michael.neumaier@medma.uni-heidelberg.de (M.N.); 4 Institute for Laboratory and Transfusion Medicine, Hospital Passau, Innstrasse 76, 94032 Passau, Germany

**Keywords:** sepsis, septic shock, organ dysfunction, lactate, S-adenosylhomocysteine

## Abstract

A common final pathway of pathogenetic mechanisms in septic organ dysfunction and death is a lack or non-utilization of oxygen. Plasma concentrations of lactate serve as surrogates for the oxygen-deficiency-induced imbalance between energy supply and demand. As S-adenosylhomocysteine (SAH) was shown to reflect tissue hypoxia, we compared the ability of SAH versus lactate to predict the progression of inflammatory and septic disease to septic organ dysfunction and death. Using univariate and multiple logistic regression, we found that SAH but not lactate, taken upon patients’ inclusion in the study close to ICU admission, significantly and independently contributed to the prediction of disease progression and death. Due to the stronger increase in SAH in relation to S-adenosylmethionine (SAM), the ratio of SAM to SAH, representing methylation potential, was significantly decreased in patients with septic organ dysfunction and non-survivors compared with SIRS/sepsis patients (2.8 (IQR 2.3–3.9) vs. 8.8 (4.9–13.8); *p* = 0.003) or survivors (4.9 (2.8–9.5) vs. 8.9 (5.1–14.3); *p* = 0.026), respectively. Thus, SAH appears to be a better contributor to the prediction of septic organ dysfunction and death than lactate in critically ill patients. As SAH is a potent inhibitor of SAM-dependent methyltransferases involved in numerous vital biochemical processes, the impairment of the SAM-to-SAH ratio in severely critically ill septic patients and non-survivors warrants further studies on the pathogenetic role of SAH in septic multiple organ failure.

## 1. Introduction

The development of organ dysfunction is a crucial clinical event during sepsis, as it directly relates to morbidity and mortality [1,2]. Organ dysfunction in sepsis is associated with multiple pathophysiological processes: An inflammatory immune response triggered by infection induces disturbances of the macro- and microcirculation [1,3]. Consecutive endothelial and microvascular dysfunction, microthrombi formation, and shunting cause ischemic hypoxia [4,5]. Furthermore, mitochondrial dysfunction leads to cytopathic hypoxia with impairment of tissue oxygen utilization as a central component of sepsis-associated organ dysfunction and failure [5,6]. Global hypoperfusion and disturbed oxygen utility induce a mismatch between oxygen supply and tissue metabolic requirements, resulting in bioenergetic failure [4,5]. To prevent cell death, a metabolic downregulation is induced [1,5,7], and energy metabolism is markedly altered in sepsis. Accordingly, lactate levels are increased, and/or lactate clearance is decreased, both of which have been reported to predict 28- or 30-day mortality in septic shock [8,9]. Although lactate is considered useful for diagnosing septic shock [2] and guidance of hemodynamic therapy [10,11], a major drawback is its failure to specifically detect tissue hypoxia, as its accumulation can be due to the acceleration of aerobic glycolysis via adrenergic stimulation [12], the inhibition of mitochondrial pyruvate dehydrogenase [13] or hampered hepatic metabolization [14]. Regarding the prediction of the progression of sepsis to septic shock, there is still no ideal biomarker that can indicate prognosis and guide treatment in sepsis [15,16].

More recently, plasma concentrations of S-adenosylhomocysteine (SAH) and S-adenosylmethionine (SAM) have been shown to be elevated in patients suffering from systemic inflammatory response syndrome (SIRS) and sepsis [17] and to predict mortality in septic patients [18]. Notably, SAH was found to be a highly sensitive marker for myocardial ischemia [19]. Hypoxia induces the breakdown of energy-rich adenine nucleotides to generate the short-lived metabolite adenosine, which itself is a well-established marker of an absolute or relative lack of oxygen [20]. The intracellularly accumulating adenosine reacts with L-homocysteine to form stable SAH catalyzed by the enzyme S-adenosylhomocysteine hydrolase as the equilibrium constant of this enzyme greatly favors the formation of SAH [21]. SAH itself is known to inhibit SAM-consuming methylation reactions since SAH is a potent inhibitor of methyltransferases [22], thereby decreasing the consumption of SAM and hence most likely increasing SAM plasma levels [23]. Thus, under pathological conditions like ischemia, SAH could be considered as a marker of tissue hypoxia, concomitantly leading to elevation of SAM.

Against this background, we hypothesized that plasma levels of SAH and SAM might serve as global markers for hypoxic bioenergetic failure during sepsis. If so, we asked if (i) SAH and SAM plasma levels determined in patients suffering from SIRS or sepsis can help to predict the progression of the disease to sepsis-associated organ dysfunction and septic shock or even death. As in previous studies [17,18], lactate levels were not analyzed in parallel with SAH or SAM in septic patients, we also asked (ii) how SAH and SAM would perform in the prediction of disease progression or death compared with lactate. The results of the present study suggest that SAH reflects tissue hypoxia better than lactate and hence might allow for improved prediction of the onset of septic organ dysfunction and death. Moreover, decreased ratios of plasma concentrations of SAM to SAH in most severely ill septic patients or non-survivors warrant further studies on the pathogenetic role of SAH in septic multiple organ dysfunction as SAH is a well-known inhibitor of SAM-dependent methyltransferases required for biosynthetic processes essential for cell proliferation and survival.

## 2. Results

### 2.1. Enrollment and Grouping of Patients by Time Course of Severity of Illness

**Figure 1:**
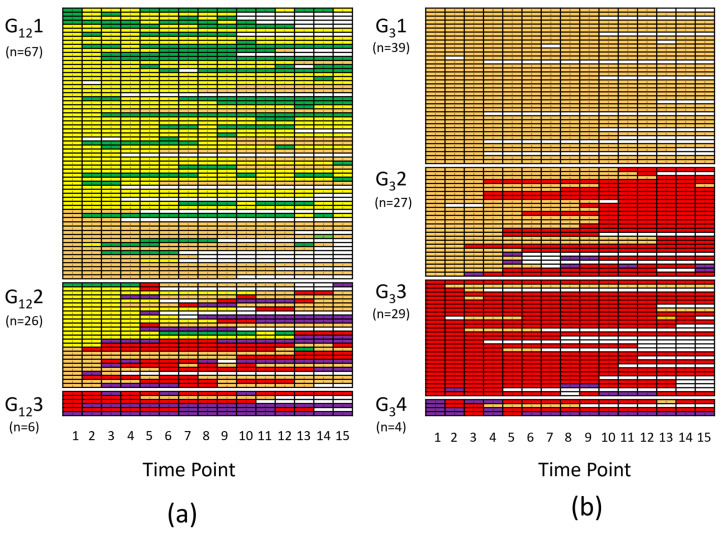
Upon admission to ICU, a total of 99 patients meeting inclusion criteria (SIRS for 48 h or sepsis for 24 h according to sepsis-1/2 definition criteria) were enrolled: (**a**) After study inclusion, patients’ status as defined by sepsis-1/2 criteria was determined every 8 h for 5 days (time points 1–15). Status of patients was classified as neither SIRS nor sepsis (status1/2 0, green), SIRS (status1/2 1, yellow), sepsis (status1/2 2, orange), severe sepsis (status1/2 3, red), and septic shock (status1/2 4, purple). Subgrouping was based on the level of septic disease severity at baseline and the development of the septic status within the study period. The status of Group 1 patients (G_12_1, n = 67) and Group 2 patients (G_12_2, n = 26) did not differ at baseline (time point 1). While the status of G_12_1 patients did not worsen within the study period (status1/2 ≤ 2), G_12_2 patients deteriorated to severe sepsis and/or septic shock (status1/2 ≥ 3, development of sepsis-associated organ dysfunction). The status of G_12_3 patients (n = 6) at time point 1 was retrospectively classified as severe sepsis and/or septic shock upon occurrence of microbiological findings. (**b**) Time course of patients’ status as defined by sepsis-3 criteria as no sepsis (status3 0, orange), sepsis (status3 1, red), and septic shock (status3 2, purple). G_3_1 (n = 39) and G_3_2 (n = 27) patients were both classified as non-septic at baseline. While G_3_1 patients stayed free of sepsis within the study period, G_3_2 patients showed progression to sepsis and/or septic shock (development of septic organ dysfunction). G_3_3 (n = 29) and G_3_4 patients (n = 4) were classified to suffer from sepsis and septic shock at baseline, respectively.

From June 2017 to June 2019, a total of 99 patients were enrolled, who fulfilled the inclusion criteria (72 patients admitted to the ICU exhibited SIRS for two consecutive days, and 27 fulfilled the criteria for sepsis according to sepsis-1/2 definition for 24 h). Patients were screened regarding the development of sepsis-associated organ dysfunction and/or septic shock within the first five days (time points 1–15) upon study inclusion according to sepsis-1/2 (Figure 1a) and sepsis-3 criteria (Figure 1b). Notably, per definition, the development of sepsis-associated organ dysfunction is represented by a shift from sepsis to severe sepsis according to sepsis-1/2 and a shift from no sepsis to sepsis according to sepsis-3. Subgrouping was based on the level of septic disease severity at baseline and the development of the septic status within the study period (Figure 1). When considering the sepsis-1/2 definition, a group of 67 patients did not worsen beyond the diagnosis of sepsis within the study period (Figure 1a, G_12_1). Hence, they did not develop a sepsis-associated organ dysfunction. By contrast, another group of 26 patients, who at baseline (=time point 1) did not differ in their status from G_12_1 patients, progressed to severe sepsis and/or septic shock (G_12_2). For a third group of six patients admitted to the ICU, who were prospectively considered to suffer from sterile SIRS accompanied by organ dysfunction or shock, microbiological findings later on revealed infection as an underlying condition, resulting in the status of severe sepsis or septic shock, respectively, already at study inclusion (G_12_3). When using the sepsis-3 definition (Figure 1b), neither G_3_1 (n = 39) nor G_3_2 (n = 27) patients had sepsis at baseline (time point 1). While G_3_1 patients remained in a non-septic state, G_3_2 patients showed progression to sepsis (representing sepsis-associated organ dysfunction) and/or septic shock within the study period. Patients of Group G_3_3 (n = 29) were classified to have sepsis and patients of Group G_3_4 (n = 4) were denoted as suffering from septic shock, already at baseline (time point 1).

### 2.2. Baseline and Clinical Characteristics of Study Participants

For all patients, demographic data, the clinical course in terms of length of ICU stay and in-hospital mortality; admitting department; chronic conditions, including Charlson comorbidity index; arterial blood gas analyses and electrolytes; clinical chemistry; hematology; vital signs; supportive therapy; and clinical scores were determined. Variables were compared at baseline (time point 1); between patients without and those with progression to sepsis-associated organ dysfunction and/or septic shock within the study period, as classified by either sepsis-1/2 or sepsis-3 definition criteria; and between in-hospital survivors and non-survivors. Table 1 displays all significant results in intergroup comparisons (variables showing no significance are summarized in Supplementary Table S1).

As can be seen in Table 1, G_12_2 patients had an almost four times higher mortality rate than G_12_1 patients (42% vs. 12%), demonstrating the clinical relevance of the progression of a patient’s status from SIRS or sepsis to severe sepsis and/or septic shock (development of sepsis-associated organ dysfunction within the study period). Notably, the status of both patient groups was not higher than sepsis at baseline and hence could not be differentiated by clinical judgment according to sepsis-1/2 definition criteria (see Figure 1a, time point 1). However, as compared to G_12_1 patients, the need for vasopressor therapy (norepinephrine and/or dobutamine), plasma urea, C-reactive protein (CRP), serum potassium, body temperature, shock index, Horovitz index, Simplified Acute Physiology Score II (SAPS II), and Sequential Organ Failure Assessment (SOFA) score were significantly higher at baseline in G_12_2 patients. Additionally, the status of patients was classified by the sepsis-3 definition criteria. Although G_3_1 and G_3_2 patients at baseline were both considered not septic (see Figure 1b, time point 1), G_3_2 patients developed sepsis or septic shock within the study period and exhibited a three times higher mortality rate than G_3_1 patients, which remained non-septic (33% vs. 10%, Table 1). At baseline, G_3_2 patients had a significantly higher need for vasopressor therapy, body temperature, respiratory rate, shock index, SpO2, and SOFA score than G_3_1 patients. Non-survivors showed significantly higher values regarding age, creatinine, urea, potassium, SAPS II, and SOFA scores than survivors.

### 2.3. Biomarkers of Energetic Failure at Baseline

Baseline plasma levels of lactate and S-adenosylmethionine (SAM) were not different between patients with (G_12_2) and without (G_12_1) progression from SIRS or sepsis to severe sepsis and/or septic shock as identified by the sepsis-1/2 definition criteria. The same was true for the comparison of patient groups with (G_3_2) and without (G_3_1) progression from non-sepsis to sepsis and/or septic shock as identified by the sepsis-3 definition criteria (Table 2). By contrast, SAH plasma levels were significantly increased in G_12_2 patients exhibiting progression of disease compared with patients who did not (*p* = 0.041). With respect to survival, all biomarkers showed statistically significantly higher values in non-survivors than in survivors (lactate *p* = 0.045; SAM *p* = 0.019; SAH *p* < 0.001). Notably, the ratio of SAM to SAH, which represents the cellular potential for the transfer of methyl groups, was significantly lower in non-survivors (*p* = 0.026; see also Supplementary Figure S1).

Regarding biomarkers at baseline, to help differentiate between patients suffering from SIRS or sepsis (G_12_1 and G_12_2) from those with severe sepsis or septic shock (G_12_3) as defined by the sepsis-1/2 definition, lactate was not statistically significant between both groups, while SAH was almost four times higher in patients with severe sepsis or septic shock (72.9 nmol/L (interquartile range (IQR), 52.1–135.0) in G_12_3 vs. 20.2 (13.5–43.2) in G_12_1 and G_12_2 combined; *p* = 0.001; Table 3). In groups defined by the sepsis-3 definition, lactate was again not significantly different, while SAM and SAH were significantly elevated in septic patients (G_3_3) as compared to non-septic patients (G_3_1 and G_3_2, *p* = 0.043 and 0.005, respectively). Interestingly, the SAM-to-SAH ratio was significantly decreased in patients with severe sepsis and/or septic shock to one-third of values determined in less severely ill patients with SIRS or sepsis (2.8 (2.3–3.9) in Group G_12_3 vs. 8.8 (4.9–13.8) in G_12_1 and G_12_2 combined; *p* = 0.003). The same effect was observed in the septic shock group defined by sepsis-3 (G_3_4, the median value of SAM/SAH was 2.8, *n* = 3) as compared to groups of non-septic (G_3_1 and G_3_2) or septic (G_3_3) patients, but it did not reach the level of statistical significance. As SAM and SAH are secreted renally, and the elevation of both compounds might be related to septic kidney dysfunction, SAM and SAH plasma concentrations were normalized to serum creatinine concentrations, still demonstrating significantly higher SAH-to-creatinine ratios in the more severely ill septic patient groups, irrespective of the sepsis definition applied (Table 3 and Supplementary Figure S2).

### 2.4. Prediction of Progression of Disease and Mortality in Univariate and Multiple Logistic Regression Analyses

For both sepsis definitions at baseline, 58 variables were tested for their usefulness to predict the progression of the disease to sepsis-associated organ dysfunction and/or septic shock within the study period and in-hospital death, respectively. Significant results in univariate testing for the progression of disease according to the sepsis-1/2 definition were obtained for CRP, urea, potassium, SOFA, SAH, body temperature, Horovitz index, shock index, SAPS II, and vasopressor therapy with decreasing order of level of significance (Table 4). For the development of sepsis-associated organ dysfunction according to the sepsis-3 definition, the order of significant univariate results was SOFA, body temperature, gender, vasopressor therapy, shock index, respiratory rate, and CRP (Table 4). With respect to the prediction of death, the statistically significant univariate variables in descending order were SAPS II, SAH, urea, creatinine, SOFA, acidosis (pH < 7.35), age, potassium, base excess, and standard bicarbonate (Table 5). Variables that were not significant for septic disease progression and death, respectively, in the univariate analysis are displayed in Supplementary Tables S2 and S3. Notably, lactate did not reach the level of statistical significance in any univariate logistic regression analyses, neither for the prediction of the progression of the disease irrespective of the type of sepsis definition used (*p* = 0.09 underlying sepsis-1/2 and *p* = 0.51 for sepsis-3) nor for the prediction of death (*p* = 0.07). Thus, lactate could not be included in the objective stepwise backward approach in multiple logistic regression analyses.

**Figure 2:**
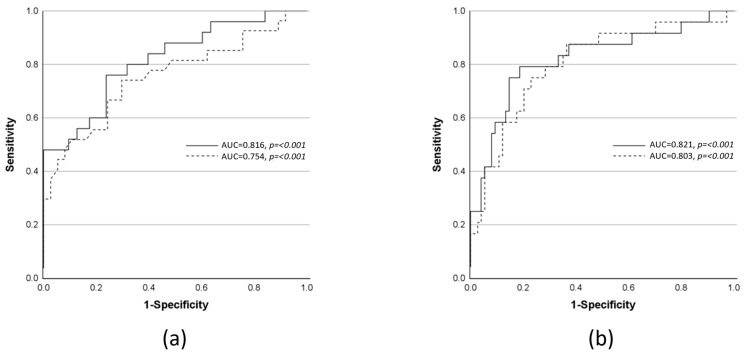
(**a**) Receiver operator characteristic (ROC) curves of the prediction of disease progression to septic organ dysfunction and/or septic shock within the study period as defined by sepsis-1/2 definition criteria (solid line). In stepwise backward multiple logistic regression C-reactive protein, body temperature, potassium, and SAH were entered into the final model. The dashed line represents the ROC curve for the prediction of disease progression to septic organ dysfunction and/or septic shock defined by sepsis-3 definition criteria. SOFA score and body temperature were entered into the final model. (**b**) ROC curves for prediction of in-hospital death based on a combination of SOFA with SAH and age (solid line) and SAPS with SAH (dashed line) as identified with stepwise backward multiple logistic regression. For further explanation, see Section 2.4. “Prediction of Progression of Disease and Mortality in Univariate and Multiple Logistic Regression Analyses”.

Final models obtained via stepwise backward multiple logistic regression analyses are summarized in Table 6, demonstrating an acceptable-to-excellent performance for the prediction of disease progression within the study period and in-hospital death. Figure 2 exemplifies the respective ROC curves for the prediction of disease progression in sepsis-1/2- and sepsis-3-defined patient groups (a) and death (b). Significant variables for the prediction of disease progression underlying the sepsis-1/2 definition were CRP, body temperature, SAH, and potassium. For the prediction of disease progression according to the sepsis-3 definition, SOFA and body temperature remained significant. For the prediction of death, SAPS II and SAH displayed independent significance in the SAPS-based approach. In the SOFA-based model, SOFA, SAH, and age were independently associated with death. Notably, SAH displayed significance in the final models of multiple logistic regression for the progression of septic disease underlying the sepsis-1/2 definition and in both models for the prediction of death. To additionally investigate the benefit of SAH regarding the prediction of outcomes, we performed AUC analyses of the final models excluding SAH. This revealed that AUROCs of models without SAH reached acceptable performance, while the inclusion of SAH led to excellent prediction of outcome (AUROC without vs. with SAH of the prediction of septic disease progression according to sepsis-1/2 definition: 0.789 vs. 0.816, the prediction of death (SAPS II-based model): 0.768 vs. 0.803, and the prediction of death (SOFA-based model): 0.787 vs. 0.821).

## 3. Discussion

The global burden of sepsis was recently estimated to be 48.9 million incident sepsis cases and 11 million sepsis-related deaths worldwide in a study from the Institute for Health Metrics and Evaluation [24]. The estimated global incidence of hospital-treated sepsis was 189 cases per 100,000 person-years along with a mortality of 27%, while for ICU-treated sepsis, the incidence was 58 cases per 100,000 person-years, with a mortality of 42% [25]. Accordingly, sepsis and its sequela still represent a major challenge for diagnosis and treatment. Specifically, upon the onset of sepsis-associated organ dysfunction, the prognosis worsens significantly, as consistently confirmed by studies based on sepsis-1/2 and sepsis-3 definition criteria [26,27]. Thus, there is a strong need for a better understanding and prediction of the development of sepsis and its progression to organ dysfunction and shock allowing for early and targeted therapeutic intervention [28]. However, there are currently no ideal biomarkers that can inform the intensivist reliably about the onset and prognosis of the disease or guide treatment in sepsis [2]. As microcirculatory hypoperfusion and mitochondrial dysfunction are thought to be among the major culprits in the pathogenesis of organ dysfunction, plasma lactate concentrations should be measured in agreement with the international guidelines for the management of sepsis and septic shock provided by the Surviving Sepsis Campaign (SSC) [29]. In fact, for adults suspected of having sepsis, measuring plasma lactate is suggested as part of the SSC 1 h bundle as an adjunctive test to modify the pre-test probability of sepsis. Additionally, lactate is suggested to be used as a target of resuscitation in the early phase of sepsis and septic shock to reduce mortality in the case of its decrease toward normal levels. Due to the low-quality evidence, these recommendations are only weak. This is even more so as there is no direct link between lactate release and tissue hypoxia [11,12,13,14,30].

In search for a metabolite that might more specifically reflect impaired tissue oxygenation, SAH was shown to detect myocardial ischemic hypoxia. In the heart, the formation of adenosine is primarily coupled to the supply–demand ratio for oxygen, and in isolated cardiomyocytes, it is substantially enhanced at critical pO2 below 3 mmHg [20]. The concentration of free intracellular adenosine in the heart was formerly assessed via the reaction of the nucleoside with L-homocysteine to form SAH catalyzed using the cytosolic SAH-hydrolase [21], with the equilibrium of this reaction being located towards the synthetic direction [31]. As SAH is a potent inhibitor of SAM-dependent methyltransferases [22,32], the accumulation of SAH under physiological conditions is controlled by maintaining levels of intracellular adenosine very low due to the phosphorylation of adenosine to AMP by high-affinity adenosine kinases. When the formation of adenosine is enhanced, e.g., as a result of ischemia and hypoxia, SAH accumulates [19]. The accumulation of SAH has also been shown in ischemic renal tissue with a long-lasting increase in this metabolite even after the restoration of perfusion [33]. Thus, these basic biochemical studies strongly suggest that, under pathological conditions induced by ischemia, the cellular formation of SAH, besides adenosine, can be considered an indicator of a lack of perfusion-dependent oxygen supply and hence tissue hypoxia.

In order to address the diagnostic and predictive value of lactate and SAH in the development of septic organ dysfunction and death in critically ill patients, the present prospective observational study was designed with the aim to identify critically ill patients with an increased probability to undergo progression in terms of the onset or worsening of sepsis. In a 6-month pilot study, senior staff intensivists daily classified the clinical status of ICU patients according to the former sepsis-1/2 definition. It was shown that patients exhibiting SIRS for at least 48 h or “simple” sepsis for 24 h had a significantly higher likelihood to develop progression to severe sepsis or septic shock within the next 5 days (see Supplementary Table S4). These findings agree with the results of Rangel-Frausto et al. [34], who were the first to show the progression from SIRS to sepsis to severe sepsis and septic shock in a prospective epidemiologic clinical study. Accordingly, critically ill patients were included in the present study if SIRS was present for 48 h or sepsis for 24 h, and they were monitored every 8 h for their clinical status during the next five days (i.e., 15 time intervals). For each sepsis definition (sepsis-1/2 and sepsis-3), a subgroup of patients remained in the baseline status (G_12_1 and G_3_1), whereas another subgroup of patients (G_12_2 and G_3_2), although starting from the same baseline status, took a different clinical course by developing septic organ dysfunction and/or shock with a major impact on survival (four times higher in-hospital mortality in the sepsis-1/2-defined group showing progression and three times higher in-hospital mortality rate in the sepsis-3-defined group with progression, Table 1). This provided the opportunity to analyze the factors predictive for the progression of the disease determined at baseline. Considering lactate as a clinically frequently used metabolic factor to indicate hypoxia, lactate was, at baseline, not significantly different between groups without or with the progression of the disease, irrespective of the sepsis definition used for patient classification. In non-survivors, compared with survivors, plasma lactate levels were significantly higher, but only to a minor degree than observed for SAH (Table 2, Supplementary Figure S1). When compared between groups with different degrees of disease severity, lactate did not show a significant difference at baseline (Table 3). In univariate analyses, to predict the progression of disease or mortality, lactate again missed the level of significance (Supplementary Tables S2 and S3). The prediction capacity of lactate for the progression from a non-septic state to sepsis or from sepsis to septic shock was, to our knowledge, not addressed before [35]. One may argue in favor of lactate that the number of patients in the present study was comparably small, but it is against this background to consider the findings on S-adenosyl compounds, especially with respect to SAH.

In contrast to lactate, already at baseline, SAH was significantly increased in sepsis-1/2-defined patients whose course of disease was complicated by septic organ dysfunction (Table 2) or death (Supplementary Figure S1) later on. SAH was also significantly higher in the more severely ill septic patient groups defined by both sepsis definitions (Table 3, Supplementary Figure S2). In univariate as well as multiple logistic regression, SAH was found to be a significant independent risk factor for the prediction of the onset of sepsis-associated organ dysfunction and/or septic shock and death, respectively (Table 6 and Figure 2). Notably, our analyses found evidence that the accumulation of SAH is unlikely to be caused solely by a decrease in renal elimination, as discussed in a previous study [18]. When SAH values were normalized for creatinine, the increase in the sepsis-1/2-defined patient group suffering from severe sepsis and/or septic shock as compared to the less severely ill patient groups remained significant (Table 3, Supplementary Figure S2). The same was true for the sepsis-3-defined sepsis compared with the non-septic group at baseline.

All these results confirm the few findings previously published on adenosine metabolites in septic patients and extend the evidence of their diagnostic value profoundly. Among those, Semmler et al. first reported significantly increased plasma concentrations of SAM and SAH in septic patients as compared to non-septic control patients with concentrations of metabolites in comparable ranges to those reported in the present study [17]. Increased SAM and SAH plasma concentrations in septic patients were corroborated by Wexler et al. and extended by observing higher levels in non-survivors compared with survivors [18]. Wang et al. focused on a SAM-related metabolite methylthioadenosine (MTA) and demonstrated that high MTA levels in plasma were associated with mortality from sepsis, while SAH and SAM approached significance (*p* = 0.05 and *p* = 0.06, respectively) [36]. All these studies focused on putative sepsis-associated disturbances of methionine metabolism and hence did not consider the possibility that SAH is likely to reflect tissue hypoxia [19,33]. Accordingly, in these studies, SAH and lactate were not compared. In the present study, baseline values of SAH were significantly higher in patients with higher morbidity as well as in non-survivors (Table 2 and Table 3, Supplementary Figures S1 and S2). Additionally, SAH baseline values were shown to be independent predictors of the progression of the disease to septic organ dysfunction and/or septic shock as well as death in multiple logistic regression models (Table 6 and Figure 2). Moreover, while the performance of the models of outcome prediction was acceptable without SAH, excellent predictive performance was achieved when SAH was included (AUROCs without SAH in the range between 0.7 and 0.8 vs. with SAH >0.8). In contrast, lactate missed statistical significance in respective analyses. The better performance of SAH shown here suggests that SAH could be a more sensitive and specific indicator of tissue hypoxia than lactate in critically ill patients.

Moreover, with the introduction of the sepsis-3 definition, emphasis was placed on the development of organ dysfunction as a diagnostic and prognostic hallmark of sepsis, thereby reminding us of the need to better understand the pathogenesis of septic organ dysfunction. Regarding the broad spectrum of methylation-dependent biochemical synthetic processes in maintaining the cell’s homeostasis, it is worth recalling that SAH is a very potent inhibitor of methyltransferases [22] required for DNA and RNA methylation, protein biosynthesis, and glutathione and catecholamine synthesis [32]. One might therefore speculate on the pathogenic role of SAH in septic organ dysfunction. In agreement with this, Semmler et al. already reported a significantly lower SAM-to-SAH ratio in septic patients [17], a finding that was confirmed (Table 2 and Table 3) and extended to non-survivors (Supplementary Figure S1) in the present study.

Taken together, significantly elevated levels of SAH in the more severely ill septic patients and non-survivors as well as its role as an independent predictor for the progression of septic disease and death warrant further studies on SAH as a novel hypoxia marker in the pathogenesis of septic organ dysfunction. With respect to the latter, a putative pathogenetic role of SAH might be argued for by its capacity to strongly inhibit SAM-dependent methyl transfer reactions in biosynthetic processes [22,32], which might become essential for survival in critically ill patients.

### Study Limitations

This was a single-center study that included a limited number of patients due to the laborious study design. Despite the limited number of study patients, the main results of the study regarding S-adenosyl compounds and lactate reached robust levels of significance and acceptable confidence intervals. Our study is limited to surgical ICU patients and patients with severe respiratory dysfunction due to the specialization of our center and thus poses the risk of impaired generalizability. Due to the initiation time of the study, the original recruitment strategy was based on the sepsis-1/2 definition. Therefore, we retrospectively additionally applied sepsis-3 criteria within the dataset, and the main results for S-adenosyl compounds and lactate proved to be robust, independent from the sepsis definition used. The group of the more severely critically ill patients with septic organ dysfunction and/or septic shock already at baseline (Group G_12_3, and consecutively G_3_3 and G_3_4) were not intended to serve as groups of comparison in the initial study design but resulted from microbiological findings that occurred with time delay. As a result, these groups are under-represented.

## 4. Materials and Methods

### 4.1. Ethics

This was a monocentric, prospective observational clinical study that was approved by the local ethics committee (see also Institutional Review Board Statement). All patients or their legal representatives gave informed consent. After recovery, previously non-self-consenting patients had the opportunity to withdraw their consent to participate in this study. Study design and reporting were based on the recommendations of the STROBE statement (https://www.strobe-statement.org/, University of Bern, Institute of Social and Preventive Medicine, Mittelstrasse 43, CH-3012 Bern, Switzerland, accessed on 13 July 2023). The study was conducted in the 24-bed intensive care unit (ICU) of the Department of Anesthesiology, Surgical Intensive Care Medicine and Pain Medicine, Mannheim University Hospital, Mannheim Medical Faculty, University of Heidelberg, from June 2017 to June 2019. In this ICU, patients from all surgical subspecialties apart from cardiac surgery are treated, and the unit is also specialized in treating respiratory dysfunction.

### 4.2. Study Population

Before study initiation, a pre-analysis was performed to increase the probability of observing a shift from SIRS/sepsis into severe sepsis/septic shock within the foreseen prospective study period of five consecutive days following patient inclusion [34]. This retrospective pre-analysis included all patients who had been treated in the ICU between May 2016 and December 2016, using the Ground Truth for Sepsis Questionnaire (GTSQ) [37]. Accordingly, the treating senior intensivists rated every patient admitted to the ICU daily at 2–3 pm by five working diagnoses in accordance with the concept of the sepsis-1/2 consensus definition. The shift from SIRS/sepsis into severe sepsis/septic shock within the five consecutive days following admission was evaluated (for details on the pre-analysis, see Supplementary Table S4).

The pre-analysis resulted in the final prospective study design with the following inclusion criteria for study patients: either continuous SIRS status for two consecutive days or sepsis according to sepsis-1/2 definition but not severe sepsis or septic shock within the first 24 h after ICU admission [38]. Exclusion criteria were age <18 years, immunosuppression, end-stage renal failure, pregnancy, need for ECMO therapy, and neurosurgical main diagnosis to exclude confounding neuroinflammation.

Upon study inclusion, the dynamic of patients’ status was assessed every 8 h for a period of 5 days, resulting in up to 15 evaluation time points. The assessment followed sepsis-1/2 definition criteria (neither SIRS nor sepsis (0), SIRS (1), sepsis (2), severe sepsis (3), or septic shock (4); for a detailed list of diagnostic sepsis-1/2 definition criteria, see Supplementary Table S5 [39,40,41]). Due to the initiation time of the study, the original prospective recruitment strategy was based on the sepsis-1/2 definition. To assess the robustness of our results regarding the current sepsis definition [2], we retrospectively additionally applied sepsis-3 criteria. Sepsis-3 criteria were applied to classify patients at evaluation time points to have no sepsis (0), sepsis (1), or septic shock (2), respectively, using an automated algorithm as described previously [2,42]. The criteria of both sepsis definitions (sepsis-1/2 and sepsis-3) were applied for all study patients at all evaluation time points, resulting in a respective label of septic status for every patient every 8 h within the study period.

The intensive care of patients followed the international guidelines of the Surviving Sepsis Campaign [10], including the use of a transpulmonary thermodilution catheter (Pulsiocath™, Pulsion Medical Systems, Munich, Germany) in hemodynamically unstable patients. The results obtained via pulse contour cardiac output monitoring (PiCCOplus™, Pulsion Medical Systems, Munich, Germany) were used to guide hemodynamic therapy. Blood gas analyses were measured routinely using a blood gas analyzer (Radiometer ABL 800 Flex, Radiometer, Willich, Germany).

### 4.3. Collection of Data and Blood Sampling

The clinical data of ICU patients were collected via the Philips Intelli Space Critical Care and Anesthesia (ICCA)™ system. Upon study inclusion, venous blood samples were taken at study inclusion (=at baseline, time point 1, ~3 pm) via a central catheter or a peripheral indwelling cannula. Following study inclusion, every eight hours (starting at 3 pm, 11 pm, and 7 am = evaluation time points), senior intensivists assigned a diagnostic label according to sepsis-1/2 and the onset of infection underlying sepsis-3 scoring [2,38], up to a total of 15 evaluations over five days, provided patients stayed on ICU. The laboratory staff had no information about patients’ condition, and likewise, the medical specialists were blinded to the laboratory results of SAH and SAM measurements. Additionally, for all study patients, clinical variables listed in Table 1 and Supplementary Table S1 were collected. The closest timely value before the referring evaluation time point was used for further analyses.

### 4.4. Determination of Plasma Concentrations of SAH and SAM

SAH and SAM plasma concentrations were determined via stable-isotope dilution LC–tandem MS/MS following the separation of analytes via pH-dependent solid-phase extraction columns containing phenylboronic acid as previously described [43]. In our protocol, interassay CVs were around 4% and 8% for SAH and 4% and 7% for SAM. The mean recoveries were 85% for SAH and 87% for SAM. Lower limits of detection were 1 and 2 nmol/L for SAH and SAM, respectively.

### 4.5. Statistical Analyses

For clinical characterization, continuous variables were compared with the *t*-test (Satterthwaite method), and categorical variables were assessed with the Chi² test. Non-normally distributed hypoxia-related variables were compared using Mann–Whitney U tests. All variables are presented as medians (interquartile range (IQR)) unless otherwise indicated. Categorical variables were expressed as numbers and percentages. Multiple logistic regression analysis was performed to identify the independent covariables (risk factors) for patients’ outcomes defined as either the progression of disease (development of sepsis-associated organ dysfunction and/or septic shock) within the study period or in-hospital death. Notably, per definition, the development of sepsis-associated organ dysfunction is represented by a shift from sepsis to severe sepsis according to sepsis-1/2 and a shift from no sepsis to sepsis according to sepsis-3. Covariables were pre-specified based on *p*-values below 0.05 from univariate logistic regression (see Table 4 and Table 5). For the outcome progression of the disease, the SOFA score, as an established score to assess organ dysfunction [44], was combined with those variables proven to be statistically significant in univariate testing, but only if variables were not related to SOFA score calculation, in order to avoid redundancy. For the outcome of in-hospital mortality, statistically significant variables in univariate testing were considered in combination with SAPS II and SOFA as established scores of prognoses in ICU patients [44,45] in two separate models, if the variables were not related to the calculation of SAPS II and SOFA scores, respectively, again to avoid redundancy. The listed covariables were sequentially removed if comparisons between the combined model and the base model were not statistically significant (the likelihood ratio test *p*-value > 0.10), starting with the covariable least strongly associated with the outcome variable (backward stepwise method). This was continued until the association with the respective outcome of all covariables remaining in the model was statistically significant. Receiver operating characteristic (ROC) curves and their area under the curve (AUC) were derived for the final models. Their discriminatory performance was evaluated with respect to sensitivity, specificity, accuracy, and diagnostic odds ratio. For this, the predicted outcome probability that maximizes the sum of sensitivity and specificity determined the optimal cutoff point. The evaluation of the performance of AUROCs followed the suggestions by Hosmer and Lemeshow [46].

Besides directly measured variables, some variables were calculated with their division, e.g., SAM by SAH for the estimation of methylation potential or SAM by creatinine and SAH by creatinine, respectively, for the normalization of renal function. No outlier or extreme values corrections and no imputation of missing data points were performed in any case of directly measured or calculated variables. All tests in this study were two-sided, and a *p*-value of less than 0.05 was considered statistically significant. Statistical analyses were performed using SAS Software V9.4 (SAS Institute, Cary, NC, USA).

## Figures and Tables

**Table 1:** Baseline and clinical characteristics of the study population.

	All (N = 99)		Groups by Sepsis-1/2 Definition *					Groups by Sepsis-3 Definition **					Groups by In-Hospital Survival				
			G_12_1 (N = 67)		G_12_2 (N = 26)			G_3_1 (N = 39)		G_3_2 (N = 27)			Survivor, S (N = 75)		Non-Survivor, NS (N = 24)		
	n		n		n		G_12_1 vs. G_12_2	n		n		G_3_1 vs. G_3_2	n		n		S vs. NS
**Demographics**																	
Age (years)	99	63 (53–76)	67	62 (53–76)	26	63 (53–78)	ns	39	61 (49–73)	27	56 (49–73)	ns	75	61 (49–74)	24	73 (60–79)	**0.002**
Male		65 (66)		43 (64)		18 (69)	ns		23 (59)		24 (89)	**0.012**		46 (61)		19 (79)	ns
**Clinical Course**																	
Pressor therapy		68 (69)		41 (61)		22 (85)	**0.037**		23 (59)		24 (89)	**0.013**		48 (64)		20 (83)	ns
ICU-LOS (days)	99	25 (16–47)	67	27 (16–46)	26	23 (18–69)	ns	39	25 (16–39)	27	37 (18–60)	ns	75	27 (18–53)	24	19 (13–34)	**0.028**
In-hospital mortality		24 (24)		8 (12)		11 (42)	**0.001**		4 (10)		9 (33)	**0.029**					
**Clinical chemistry at baseline**																	
Creatinine (mg/dL)	96	0.98 (0.73–1.50)	64	0.88 (0.68–1.29)	26	1.19 (0.79–1.68)	ns	38	0.80 (0.66–1.22)	27	1.10 (0.75–1.38)	ns	72	0.88 (0.68–1.26)	24	1.72 (1.05–2.72)	**0.003**
Urea (mg/dL)	96	45.1 (33.4–63.8)	64	41.9 (29.3–58.1)	26	51.4 (41.8–76.8)	**0.015**	38	37.5 (27.1–56.1)	27	45.2 (31.7–55.4)	ns	72	42.2 (30.8–55.8)	24	60.1 (43.7–81.7)	**0.007**
CRP (mg/dL)	96	150 (90–218)	64	132 (80–176)	26	221 (141–253)	**0.001**	38	124 (59–171)	27	150 (92–244)	ns	72	152 (91–220)	24	146 (86–216)	ns
K+ (mmol/L)	98	4.1 (3.8–4.3)	66	4.0 (3.8–4.2)	26	4.3 (4.1–4.5)	**0.012**	38	4.0 (3.8–4.2)	27	4.2 (4.0–4.4)	ns	74	4.0 (3.8–4.2)	24	4.3 (4.1–4.5)	**0.041**
**Vital signs at baseline**																	
Temperature (°C)	96	37.1 (36.8–37.7)	65	37.0 (36.6–37.6)	25	37.4 (37.0–37.9)	**0.016**	37	37.1 (36.8–37.5)	27	37.4 (37.1–37.9)	**0.006**	72	37.1 (36.8–37.7)	24	37.1 (36.8–37.6)	ns
Resp. rate (1/min)	98	19 (16–22)	66	18 (15–22)	26	20 (18–22)	ns	38	17 (15–20)	27	21 (18–24)	**0.006**	74	19 (16–22)	24	20 (16–25)	ns
Shock index	98	0.70 (0.61–0.88)	66	0.68 (0.58–0.85)	26	0.79 (0.62–0.90)	**0.039**	38	0.68 (0.59–0.86)	27	0.82 (0.65–0.93)	**0.024**	74	0.71 (0.60–0.87)	24	0.69 (0.62–0.95)	ns
SpO2 (%)	98	98 (95–100)	66	99 (96–100)	26	97 (96–99)	ns	38	99 (97–100)	27	97 (96–99)	**0.035**	74	99 (96–100)	24	97 (95–98)	ns
Horovitz (mmHg)	98	280 (220–343)	66	302 (234–362)	26	235 (199–320)	**0.010**	38	303 (244–362)	27	266 (220–326)	ns	74	301 (229–347)	24	218 (185–298)	ns
**Clinical scores at baseline**																	
SAPS II	98	35 (28–43)	66	33 (25–40)	26	37 (32–45)	**0.039**	38	33 (25–39)	27	33 (28–38)	ns	74	33 (25–39)	24	42 (36–51)	**<0.001**
SOFA	99	8 (5–11)	67	7 (5–10)	26	9 (7–13)	**0.024**	39	8 (5–11)	27	11 (8–13)	**0.004**	75	8 (5–10)	24	11 (7–13)	**0.013**

* Sepsis-1/2 defined patient groups without (G_12_1) and with (G_12_2) progression from SIRS or sepsis to severe sepsis and/or septic shock (development of septic organ dysfunction (SOD) within the study period). ** Sepsis-3 defined groups remaining sepsis-free (G_3_1) or progress to sepsis (G_3_2, development of SOD). Results as median (interquartile range, IQR) or number (%); significant results in bold. Abbreviations: N = number of patients included; n = number of determinations for variable; ns = not significant; LOS = length of stay; CRP = C-reactive protein; K+ = potassium; resp = respiratory; SpO2 = peripheral capillary oxygen saturation; Horovitz = Horovitz index; SAPS = Simplified Acute Physiology Score; SOFA = Sequential Organ Failure Assessment.

**Table 2:** Lactate and S-adenosyl compounds at baseline for patient groups of different clinical outcomes *.

	Groups by Sepsis-1/2 Definition					Groups by Sepsis-3 Definition					Groups by In-Hospital Survival				
	G_12_1 (N = 67)		G_12_2 (N = 26)			G_3_1 (N = 39)		G_3_2 (N = 27)			Survivor (S) (N = 75)		Non-Survivor (NS) (N = 24)		
	n		n		G_12_1 vs. G_12_2	n		n		G_3_1 vs. G_3_2	n		n		S vs. NS
**Lactate (mmol/L)**	66	0.9 (0.7–1.5)	26	1.1 (0.8–1.6)	ns	38	1.0 (0.8–1.6)	27	0.9 (0.6–1.5)	ns	74	0.9 (0.7–1.5)	24	1.1 (0.9–1.7)	**0.045**
**SAM (nmol/L)**	66	161 (123–238)	23	195.3 (130–281)	ns	38	155 (121–238)	26	170 (130–230)	ns	72	158 (123–234)	23	207 (148–310)	**0.019**
**SAH (nmol/L)**	67	18.5 (12.4–33.7)	26	29.2 (16.5–58.2)	**0.041**	39	18.5 (11.8–33.7)	27	18.0 (13.4–43.2)	ns	75	18.6 (13.4–32.0)	24	47.6 (23.4–91.3)	**<0.001**
**SAM/SAH**	66	9.2 (5.0–14.5)	23	8.0 (3.6–12.6)	ns	38	8.4 (4.0–14.5)	26	8.0 (5.2–13.7)	ns	72	8.9 (5.1–14.3)	23	4.9 (2.8–9.5)	**0.026**
**SAM/Crea**	63	165 (109–236)	23	151 (116–295)	ns	37	169 (118–240)	26	161 (109–210)	ns	69	165 (114–240)	23	138 (78–235)	ns
**SAH/Crea**	64	20.4 (15.4–31.3)	26	29.0 (17.1–36.1)	ns	38	20.0 (15.4–31.6)	27	20.8 (13.7–31.8)	ns	72	20.7 (15.6–31.6)	24	31.7 (17.7–40.8)	ns

* Sepsis-1/2 defined patients in groups G_12_1 and G_12_2 without or with progression from SIRS or sepsis to severe sepsis and/or septic shock (development of septic organ dysfunction within the study period). Sepsis-3 defined patients in groups G_3_1 and G_3_2 who remained sepsis-free or progressed to sepsis and/or septic shock (development of septic organ dysfunction) within the study period. Results are displayed as median (interquartile range, IQR); significant results are highlighted in bold. Abbreviations: N = number of patients included; n = number of determinations for respective variable; ns = not significant; SAM = S-adenosylmethionine; SAH = S-adenosylhomocysteine; Crea = creatinine. Data of groups were compared using Mann–Whitney U test.

**Table 3:** Lactate and S-adenosyl compounds for patient groups with different degrees of severity of critical illness at baseline *.

	Groups by Sepsis-1/2 Definition					Groups by Sepsis-3 Definition							
	G_12_1 + G_12_2 (N = 93)		G_12_3 (N = 6)			G_3_1 + G_3_2 (N = 66)		G_3_3 (N = 29)		G_3_3 + G_3_4 (N = 33)			
	n		n		G_12_1 + G_12_2 vs. G_12_3	n		n		n		G_3_1 + G_3_2 vs. G_3_3	G_3_1 + G_3_2 vs. G_3_3 + G_3_4
**Lactate (mmol/L)**	92	0.9 (0.7–1.6)	6	1.3 (1.1–2.2)	ns	65	0.9 (0.7–1.5)	29	0.9 (0.7–1.2)	33	1.0 (0.7–1.6)	ns	ns
**SAM (nmol/L)**	89	174 (127–257)	6	197 (168–263)	ns	64	158 (127–238)	28	208 (144–334)	31	197 (140–331)	**0.043**	ns
**SAH (nmol/L)**	93	20.2 (13.5–43.2)	6	72.9 (52.1–135.0)	**0.001**	66	18.3 (12.4–42.5)	29	29.0 (20.0–77.2)	33	29.7 (20.5–77.2)	**0.005**	**0.003**
**SAM/SAH**	89	8.8 (4.9–13.8)	6	2.8 (2.3–3.9)	**0.003**	64	8.0 (4.5–14.3)	28	8.8 (3.7–11.0)	31	8.8 (2.8–10.1)	ns	ns
**SAM/Crea**	86	165 (114–240)	6	93 (69–163)	ns	63	165 (114–229)	26	164 (69–341)	29	153 (75–292)	ns	ns
**SAH/Crea**	90	21.4 (15.4–35.3)	6	44.9 (26.9–57.9)	**0.008**	65	20.6 (15.4–31.7)	27	30.1 (17.4–39.4)	31	30.1 (17.4–39.5)	**0.022**	**0.011**

* Patient groups defined with sepsis-1/2: Both groups had SIRS or sepsis at baseline. While Group G_12_1 exhibited no progression of disease, Group G_12_2 designated patients who developed septic organ dysfunction and/or septic shock within the study period. Patient groups defined with sepsis-3: While G_3_1 designated patients who remained sepsis-free, patients in Group G_3_2 developed septic organ dysfunction and/or septic shock within the study period. G_12_3: sepsis-1/2-defined patients with severe sepsis or septic shock; G_3_3: sepsis-3-defined patients with sepsis, G_3_4: sepsis-3-defined patients with septic shock. Results are displayed as median (interquartile range, IQR); significant results are highlighted in bold. Abbreviations: N = number of patients included; n = number of determinations for respective variable; ns = not significant; SAM = S-adenosylmethionine; SAH = S-adenosylhomocysteine; Crea = creatinine. Data of groups were compared using Mann–Whitney U test.

**Table 4:** Univariate testing for prediction of progression of disease *.

Sepsis-1/2 Definition		Sepsis-3 Definition	
Variable	*p*-Value	Variable	*p*-Value
CRP (mg/L)	0.002	SOFA	0.006
Urea (mg/dL)	0.011	Temperature (°C)	0.010
Potassium (mmol/L)	0.017	Gender	0.013
SOFA	0.018	Vasopressor therapy	0.018
SAH (nmol/L)	0.019	Shock index	0.028
Temperature (°C)	0.024	Respiratory rate (1/min)	0.029
Horovitz index (mmHg)	0.025	CRP (mg/L)	0.041
Shock index	0.028		
SAPS II	0.041		
Vasopressor therapy	0.044		

* Significant variables in univariate testing for prediction of progression of disease within the study period in descending order of *p*-values based on applied sepsis definition; non-significant variables are displayed in Supplementary Table S2. Abbreviations: CRP = C-reactive protein; SOFA = Sequential Organ Failure Assessment; SAH = S-adenosylhomocysteine; SAPS = Simplified Acute Physiology Score.

**Table 5:** Univariate testing for prediction of in-hospital death *.

Variable	*p*-Value
SAPS II	<0.001
SAH (nmol/L)	<0.001
Urea (mg/dL)	0.002
Creatinine (mg/dL)	0.005
SOFA	0.008
Acidosis (pH < 7.35)	0.010
Age (years)	0.013
Potassium (mmol/L)	0.026
Standard base excess (mmol/L)	0.029
Standard bicarbonate (mmol/L)	0.030

* Significant variables in univariate testing for prediction of death in descending order of *p*-values; non-significant variables are displayed in Supplementary Table S3. Abbreviations: SAPS = Simplified Acute Physiology Score; SAH = S-adenosylhomocysteine; SOFA = Sequential Organ Failure Assessment.

**Table 6:** Multiple logistic regression models based on baseline parameters for prediction of progression of septic disease or in-hospital death *.

Predicted Outcome	N	AUROC (*p*-Value)	TP	FP	FN	TN	Sensitivity (%) (95% CI)	Specificity (%) (95% CI)	Diagnostic OR (95% CI)	Accuracy (%)	Model Parameters	OR (95% CI)	*p*-Value of OR
**Progression from SIRS or sepsis to severe sepsis or septic shock** **(sepsis-1/2 definition)**	88	0.816 **(<0.001)**	19	15	6	48	76 (57–89)	76 (64–85)	10.13 (3.42–30.01)	76.1	CRP	1.01 (1.00–1.02)	**0.005**
											Temperature	4.01 (1.48–10.86)	**0.006**
											K+	5.75 (1.31–25.21)	**0.020**
											SAH	1.02 (1.00–1.04)	**0.033**
**Progression from non-sepsis to sepsis or septic shock** **(sepsis-3 definition)**	64	0.754 **(<0.001)**	20	11	7	26	74 (55–89)	70 (54–83)	6.75 (2.22–20.55)	71.9	SOFA	1.24 (1.04–1.48)	**0.018**
											Temperature	3.37 (1.23–9.27)	**0.019**
**Progression to death** **(SAPS II-based model)**	98	0.803 **(<0.001)**	18	17	6	57	75 (55–88)	77 (66–85)	10.06 (3.45–29.35)	76.5	SAPS II	1.08 (1.01–1.15)	**0.021**
											SAH	1.02 (1.00–1.04)	**0.030**
**Progression to death** **(SOFA-based model)**	99	0.821 **(<0.001)**	19	14	5	61	79 (60–91)	81 (71–89)	16.56 (5.28–51.96)	80.8	SOFA	1.25 (1.05–1.50)	**0.015**
											SAH	1.02 (1.00–1.04)	**0.014**
											Age	1.06 (1.01–1.11)	**0.011**

* For each model obtained with stepwise backward multiple logistic regression, the predicted outcome probability that maximized the sum of sensitivity and specificity served as the threshold for patient classification and model performance measures. Significant results in bold. For further explanation, see Section 2.4. Abbreviations: N = number of observations; AUROC = area under the receiver operating characteristic curve; TP = true-positive cases; FP = false-positive cases; FN = false-negative cases; TN = true-negative cases; CI = confidence interval; OR = odds ratio; SIRS = systemic inflammatory response syndrome; CRP = C-reactive protein; K+ = potassium; SAH = S-adenosylhomocysteine; SOFA = Sequential Organ Failure Assessment; SAPS = Simplified Acute Physiology Score.

## Data Availability

The datasets generated and analyzed during the current study are not publicly available due to patient privacy but are available from the corresponding author upon reasonable request.

## Supplementary Materials

The following supporting information can be downloaded at: https://www.mdpi.com/article/10.3390/ijms241612600/s1.
