# Supplementary material for: S-Adenosylhomocysteine Is a Useful Metabolic Factor in the Early Prediction of Septic Disease Progression and Death in Critically Ill Patients: A Prospective Cohort Study
Source: Int J Mol Sci. 2023 Aug 9;24(16):12600. doi: 10.3390/ijms241612600 (PMC10454796; doi:10.3390/ijms241612600)
Supplement: Supplementary file 1 [file ijms-24-12600-s001.zip › Supplementary Figure S1.pdf]

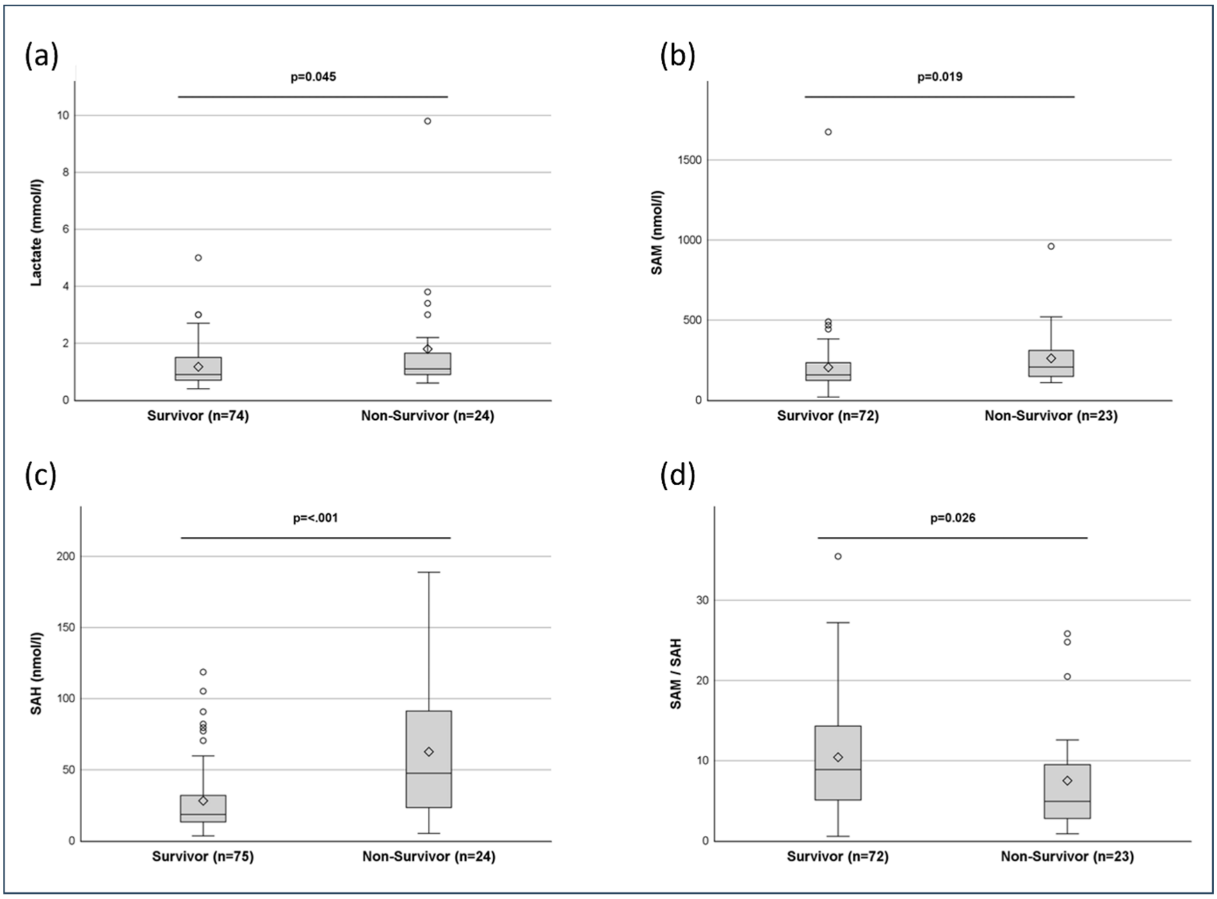

Supplementary Figure S1. Plasma concentrations of lactate (a), S-adenosylmethionine (SAM, (b)), S-adenosylhomocysteine (SAH, (c)), and ratio of SAM to SAH (d) in survivors and non-survivors at baseline. Data are represented by box plots, with upper and lower line of the box representing the 25th and 75th percentile, the horizontal line and the diamond in the box show the median and mean value, respectively. SAM-to-SAH ratio was calculated by division of SAM by SAH values. n gives the number of patients measured. Data of groups were compared by Mann-Whitney U test.
