# Supplementary material for: S-Adenosylhomocysteine Is a Useful Metabolic Factor in the Early Prediction of Septic Disease Progression and Death in Critically Ill Patients: A Prospective Cohort Study
Source: Int J Mol Sci. 2023 Aug 9;24(16):12600. doi: 10.3390/ijms241612600 (PMC10454796; doi:10.3390/ijms241612600)
Supplement: Supplementary file 1 [file ijms-24-12600-s001.zip › Supplementary Figure S2.pdf]

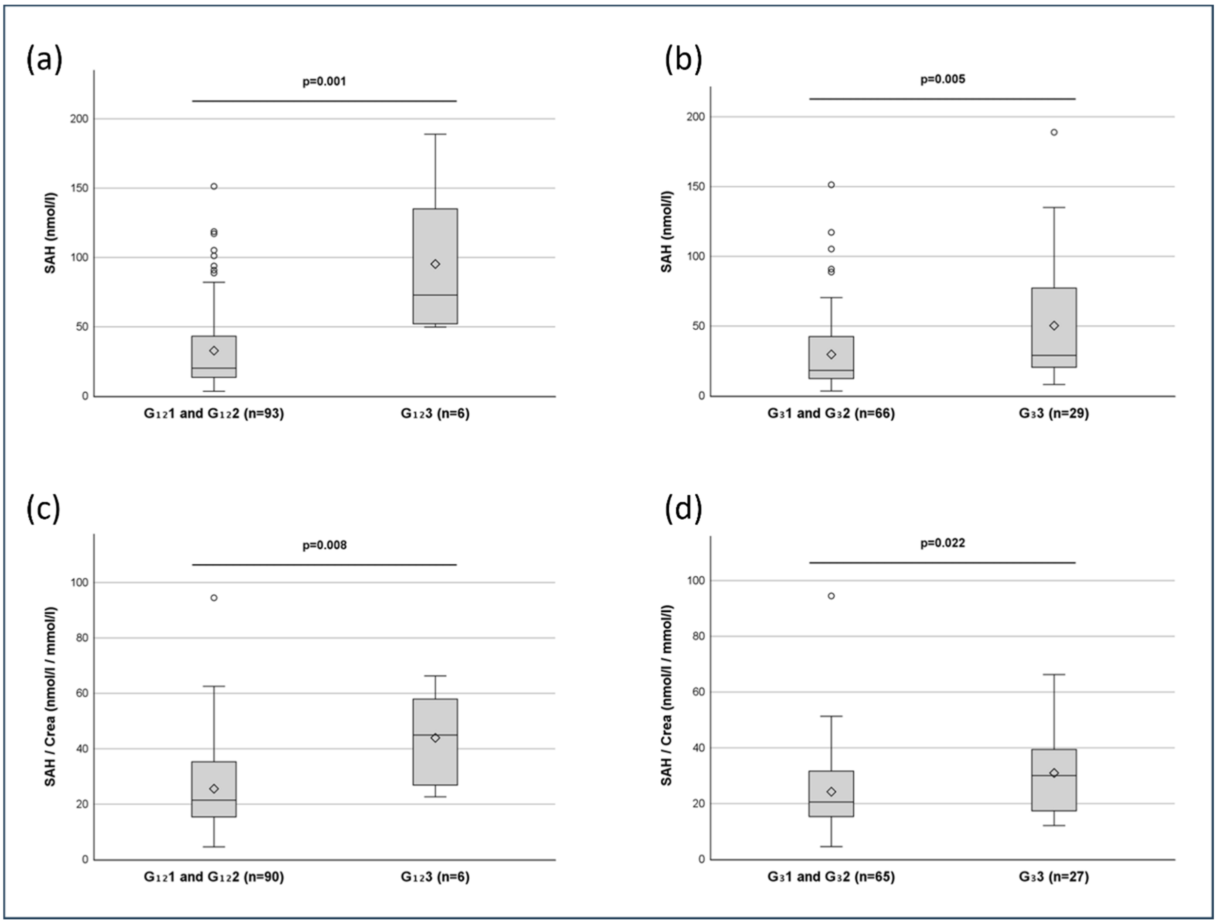

Supplementary Figure S2. Plasma concentrations of S-adenosylhomocysteine (SAH, (a)) and ratio of SAH to Creatinine (b) at baseline in sepsis-1/2 defined groups with SIRS or 'simple' sepsis ($G_{12}1$ combined with $G_{12}2$) compared to the group of patients suffering from severe sepsis or septic shock ($G_{12}3$). Respective results are shown for sepsis-3 defined patient groups ((c) and (d)) for intergroup comparison of patients without ($G_{3}1$ combined with $G_{3}2$) and with sepsis ($G_{3}3$), at baseline. Data are represented by box plots, with upper and lower line of the box representing the 25th and 75th percentile, the horizontal line and the diamond in the box show the median and mean value, respectively. SAH-to-Crea ratio was calculated by division of SAH by creatinine values. n gives the number of patients measured. Data of groups were compared by Mann-Whitney U test.
