# Supplementary material for: S-Adenosylhomocysteine Is a Useful Metabolic Factor in the Early Prediction of Septic Disease Progression and Death in Critically Ill Patients: A Prospective Cohort Study
Source: Int J Mol Sci. 2023 Aug 9;24(16):12600. doi: 10.3390/ijms241612600 (PMC10454796; doi:10.3390/ijms241612600)
Supplement: Supplementary file 1 [file ijms-24-12600-s001.zip › Supplementary Table S1.pdf]

Table S1. Baseline and clinical characteristics of the study population represented by variables which in intergroup comparisons were tested non-significant*.

	All (N=99)		Groups by sepsis-1/2 definition					Groups by sepsis-3 definition					Groups by survival				
			$G_{12}1$ (N=67)		$G_{12}2$ (N=26)			$G_{3}1$ (N=39)		$G_{3}2$ (N=27)			Survivor, S (N=75)		Non-Survivor, NS (N=24)		
	n		n		n		$G_{12}1$ vs $G_{12}2$	n		n		$G_{3}1$ vs $G_{3}2$	n		n		S vs NS
**Admitting Department**																	
Surgery		39 (39)		22 (33)		14 (54)	ns		13 (33)		13 (48)	ns		27 (36)		12 (50)	ns
Orthopedics and trauma center		27 (27)		16 (24)		10 (39)	ns		8 (21)		11 (41)	ns		23 (31)		4 (17)	ns
Neurosurgery		24 (24)		18 (27)		4 (15)			15 (39)		5 (19)	ns		17 (23)		7 (29)	ns
Otorhino-laryngology		9 (9)		8 (12)		1 (4)			4 (10)		1 (4)	ns		8 (11)		1 (4)	
Others		10 (10)		7 (10)		3 (12)			3 (8)		1 (4)	ns		8 (11)		2 (8)	
**Chronic conditions**																	
Diabetes		18 (18)		14 (21)		3 (12)	ns		9 (23)		2 (7)	ns		15 (20)		3 (13)	ns
Cardiovascular diseases		43 (43)		26 (39)		13 (50)	ns		17 (44)		10 (37)	ns		29 (39)		14 (58)	ns
Respiratory diseases		5 (5)		2 (3)		2 (8)					1 (4)	ns		2 (3)		3 (13)	
Alcoholism		6 (6)		5 (8)		1 (4)			3 (8)		2 (7)	ns		6 (8)			
Charlson Comorbidity Index	99	1 (1-3)	67	1 (1-3)		1 (1-3)	ns	39	1 (1-3)	27	1 (0-3)	ns		1 (1-3)		1 (1-4)	ns
**BGA and electrolytes**																	
PO2 [mmHg]	98	89 (77-103)	66	91 (78-104)	26	81 (78-96)	ns	38	92 (84-106)	27	88 (78-110)	ns	74	90 (78-103)	24	81 (73-108)	ns

PCO2 [mmHg]	98	43 (39-47)	66	43 (39-48)	26	42 (39-47)	ns	38	45 (39-48)	27	44 (42-51)	ns	74	43 (39-47)	24	42 (36-48)	ns
ScvO2 [%]	73	75 (71-78)	43	75 (71-79)	24	73 (68-77)	ns	27	75 (71-78)	22	74 (71-79)	ns	52	75 (70-78)	21	74 (71-77)	ns
pH	98	7.42 (7.37-7.45)	66	7.42 (7.38-7.45)	26	7.40 (7.35-7.45)	ns	38	7.42 (7.40-7.45)	27	7.39 (7.36-7.42)	ns	74	7.42 (7.38-7.45)	24	7.41 (7.35-7.45)	ns
HCO3-stand [mmol/L]	98	27.5 (25.3-29.7)	66	27.9 (26.1-29.5)	26	27.2 (24.1-30.1)	ns	38	27.5 (26.5-29.9)	27	28.0 (25.4-29.8)	ns	74	28.0 (25.7-29.7)	24	26.4 (22.1-29.3)	ns
BEstand [mmol/L]	98	3.3 (1.1-5.4)	66	3.5 (2.0-5.4)	26	2.5 (-0.4-5.1)	ns	38	3.3 (2.2-5.5)	27	3.4 (1.1-4.8)	ns	74	3.8 (1.8-5.4)	24	2.2 (-2.2-5.0)	ns
Na+ [mmol/L]	98	141 (137-143)	66	141 (137-143)	26	141 (137-145)	ns	38	141 (138-143)	27	141 (137-143)	ns	74	141 (137-142)	24	143 (138-145)	ns
Cl- [mmol/L]	98	107 (105-110)	66	107 (105-109)	26	109 (106-111)	ns	38	107 (105-109)	27	109 (106-111)	ns	74	107 (105-109)	24	109 (105-111)	ns
ionized Ca++ [mmol/L]	98	1.12 (1.07-1.16)	66	1.12 (1.09-1.17)	26	1.10 (1.05-1.13)	ns	38	1.13 (1.10-1.19)	27	1.10 (1.07-1.15)	ns	74	1.12 (1.08-1.16)	24	1.10 (1.07-1.21)	ns
SIDa [mmol/L]	97	38.3 (35.1-40.0)	66	38.4 (35.7-40.1)	26	37.3(33.9-39.5)	ns	38	38.4 (35.6-40.1)	27	37.2 (33.9-39.2)	ns	74	37.7 (35.1-39.9)	23	39.0 (34.5-40.0)	ns
**Clinical chemistry**																	
Glucose [mg/dL]	98	127 (110-167)	66	128.5 (114-170)	26	121 (106-158)	ns	38	133 (114-175)	27	129 (110-158)	ns	74	127.5 (111-167)	24	125 (107-163)	ns
Bilirubin [mg/dL]	93	0.61 (0.35-0.94)	62	0.49 (0.32-0.85)	25	0.81 (0.44-1.19)	ns	37	0.66 (0.32-0.93)	26	0.68 (0.45-0.97)	ns	71	0.61 (0.34-0.97)	22	0.63 (0.41-0.94)	ns
AST [U/L]	90	43 (27-90)	60	42 (26-79.5)	25	65 (31-111)	ns	35	42 (30-101)	26	72.5 (31-143)	ns	69	42 (29-93)	21	48 (26-78)	ns
ALT [U/L]	92	37 (20-85)	61	36 (19-64)	25	44 (21-106)	ns	36	36 (19.5-89)	26	41 (21-117)	ns	70	39 (20-88)	22	28 (20-82)	ns
Lipase [U/L]	90	86 (61-207)	59	87 (56-200)	25	82 (61-163)	ns	36	104 (64-224)	25	76 (51-154)	ns	69	87 (61-193)	21	82 (61-357)	ns
PCT [µg/L]	69	0.63 (0.20-2.29)	40	0.40 (0.19-1.55)	23	0.99 (0.22-2.38)	ns	23	0.39 (0.19-1.24)	19	0.98 (0.19-1.67)	ns	52	0.59 (0.19-2.13)	17	0.90 (0.32-2.29)	ns
**Clinical scores at baseline**																	
Hb [g/dL]	98	8.9 (8.1-10.1)	66	8.8 (8.1-10)	26	8.9 (8.1-10)	ns	38	8.6 (8.1-9.2)	27	8.9 (7.9-10)	ns	74	8.9 (7.9-10.1)	24	8.9 (8.4-9.7)	ns
WBC [10E9/L]	96	11.5 (8.3-14.3)	64	11.1 (8.9-13.6)	26	11.4 (6.7-15.4)	ns	38	11.9 (9.1-14.1)	27	10.6 (6.7-13.0)	ns	72	11.9 (9.0-14.3)	24	10.4 (5.9-14.3)	ns
INR	96	1.07 (1.00-1.12)	64	1.06 (1.00-1.12)	26	1.07 (1.00-1.12)	ns	38	1.07 (1.01-1.11)	27	1.02 (0.99-1.12)	ns	72	1.06 (1.00-1.11)	24	1.10 (1.00-1.19)	ns
Thrombocytes [10E9/L]	96	162 (106-250)	64	157 (110-232)	26	175 (105-252)	ns	38	139 (95-201)	27	142 (104-213)	ns	72	166 (114-256)	24	137 (95-245)	ns
Basophils [%]	72	0.2 (0.1-0.3)	49	0.2 (0.1-0.2)	17	0.2 (0.1-0.3)	ns	31	0.2 (0.1-0.2)	20	0.2 (0.1-0.3)	ns	55	0.2 (0.1-0.3)	17	0.2 (0.1-0.3)	ns
Eosinophils [%]	72	0.3 (0.1-0.9)	49	0.3 (0.1-0.8)	17	0.5 (0.0-1.4)	ns	31	0.1 (0.0-0.6)	20	0.5 (0.1-1.2)	ns	55	0.3 (0.1-1.0)	17	0.2 (0.0-0.6)	ns

Neutrophils [%]	72	82.9 (77.9-87.4)	49	83.7 (77.9-87.7)	17	82.7 (78.0-85.8)	ns	31	83.8 (75.9-87.2)	20	81.1 (76.0-83.8)	ns	55	83.0 (77.8-87.5)	17	82.7 (79.9-86.2)	ns
Lymphocytes [%]	72	7.9 (5.4-11.5)	49	7.4 (5.5-11.9)	17	9 (6.3-10.9)	ns	31	9.2 (6.0-12.4)	20	9.8 (6.8-13.6)	ns	55	9.0 (5.5-11.6)	17	7.6 (4.7-11.4)	ns
Monocytes [%]	72	7.3 (5.8-10.1)	49	7.2 (5.2-10.5)	17	7.5 (6.5-9.4)	ns	31	7.0 (5.2-11.4)	20	7.6 (6.3-9.9)	ns	55	7.2 (5.7-10.1)	17	7.3 (6.5-10.0)	ns
**Vital signs**																	
Heart rate [1/min]	98	90 (75-101)	66	86 (74-99)	26	93 (79-110)	ns	38	88 (77-97)	27	99 (75-113)	ns	74	90 (77-99)	24	83 (70-108)	ns
Syst BP [mmHg]	98	123 (109-134)	66	124 (111-138)	26	119 (107-131)	ns	38	124 (109-140)	27	116 (106-129)	ns	74	124 (113-135)	24	119 (106-132)	ns
Diast BP [mmHg]	98	59 (53-68)	66	61 (53-68)	26	57 (54-66)	ns	38	61 (52-68)	27	60 (56-71)	ns	74	60 (53-68)	24	57 (51-66)	ns
MAP [mmHg]	98	80 (72-90)	66	82 (75-93)	26	77 (73-87)	ns	38	82 (75-95)	27	81 (71-90)	ns	74	82 (73-91)	24	76 (68-86)	ns
**Supportive Therapy**																	
Mechanical ventilation		86 (87)		57 (85)		23 (89)	ns		34 (87)		26 (96)	ns		64 (85)		22 (92)	ns
FiO2	92	0.30 (0.30-0.40)	61	0.30 (0.30-0.35)	25	0.35 (0.30-0.40)	ns	37	0.30 (0.30-0.35)	27	0.30 (0.30-0.40)	ns	69	0.30 (0.30-0.35)	23	0.35 (0.30-0.5)	ns
Volume balance [ml]	98	15 (-64-79)	66	14 (-78-73)	26	19 (-47-82)	ns	38	20 (-64-86)	27	11 (-25-64)	ns	74	14 (-64-82)	24	18 (-82-70)	ns
**Clinical Scores**																	
RASS**	97	-3 (-5-0)	65	-3 (-5-0)	26	-4 (-5-0)	ns	38	-3 (-5-0)	27	-4 (-5--3)	ns	74	-3 (-5--1)	23	-1 (-5-0)	ns
TISS	98	18 (10-22)	66	15 (10-22)	26	18 (13-23)	ns	38	18 (10-22)	27	22 (15-23)	ns	74	18 (10-22)	24	22 (11.5-25)	ns

*Sepsis-1/2 defined patient groups $G_{12}1$ and $G_{12}2$ designate patients without and with progression from SIRS or sepsis to severe sepsis or septic shock (development of septic organ dysfunction). Sepsis-3 defined groups $G_{3}1$ and $G_{3}2$ designate patients who remain sepsis free or progress to sepsis (development of septic organ dysfunction). Results displayed as median (interquartile range, IQR) or number (%); ** °-mode for RASS calculation; Abbreviations: N=number of patients included; n=number of determinations for respective variable; ns=not significant; PO2=partial pressure of oxygen; PCO2=partial pressure of carbon dioxide; ScvO2=central venous oxygen saturation; HCO3-stand=standard bicarbonate; BEstand=standard base excess; Na+=sodium; Cl-=chloride; Ca++=calcium; SIDa=strong ion difference apparent; AST=aspartate aminotransaminase; ALT=alanine aminotransaminase; PCT=procalcitonin; Hb=hemoglobin; WBC=white blood cell count; INR=international normalized ratio; Syst=systolic; BP=blood pressure; Diast=diastolic; MAP=mean arterial pressure; FiO2=fraction of inspired oxygen; RASS=Richmond agitation sedation scale; TISS=therapeutic intervention score scale.
