# Supplementary material for: S-Adenosylhomocysteine Is a Useful Metabolic Factor in the Early Prediction of Septic Disease Progression and Death in Critically Ill Patients: A Prospective Cohort Study
Source: Int J Mol Sci. 2023 Aug 9;24(16):12600. doi: 10.3390/ijms241612600 (PMC10454796; doi:10.3390/ijms241612600)
Supplement: Supplementary file 1 [file ijms-24-12600-s001.zip › Supplementary Table S2.pdf]

Table S2. Univariate analysis for prediction of progression of disease for variables which were tested non-significant *.

Sepsis-1/2 definition		Sepsis-3 definition	
**Variable**	**p-value**	**Variable**	**p-value**
Heart rate [1/min]	0.054	Heart rate [1/min]	0.062
Ionized Calcium++ [mmol/L]	0.056	Ionized Calcium++ [mmol/L]	0.074
AST [U/L]	0.063	RASS	0.078
Volume balance [mL]	0.070	Horovitz Index [mmHg]	0.090
TISS	0.075	Systolic blood pressure [mmHg]	0.110
Lactate [mmol/L]	0.092	Diabetes	0.110
Bilirubin [mg/dL]	0.105	TISS	0.146
ScvO2 [%]	0.105	SpO2 [%]	0.151
Mean arterial pressure [mmHg]	0.150	Creatinine [mg/dL]	0.168
Acidosis (pH<7.35)	0.156	Mean arterial pressure [mmHg]	0.194
Creatinine [mg/dL]	0.168	BEstand [mmol/L]	0.203
PO2 [mmHg]	0.181	AST [U/L]	0.240
BEstand [mmol/L]	0.182	SIDa [mmol/L]	0.248
HCO3stand [mmol/L]	0.201	Glucose [mg/dL]	0.277
Systolic blood pressure [mmHg]	0.211	HCO3stand [mmol/L]	0.279
Basophils [%]	0.238	Urea [mg/dL]	0.294
Respiratory rate [1/min]	0.240	K+ [mmol/L]	0.295
ALT [U/L]	0.257	Hb [g/dL]	0.298
Diabetes	0.302	Lymphocytes [%]	0.318
SIDa [mmol/L]	0.312	SAH [nmol/l]	0.324
Cardiovascular diseases	0.328	Mechanical ventilation	0.330
Respiratory diseases	0.333	Basophils [%]	0.333
Age [yrs]	0.351	Volume balance [mL]	0.336
Glucose [mg/dL]	0.383	Charlson Comorbidity Index	0.349
Chloride- [mmol/L]	0.418	PO2 [mmHg]	0.362
SpO2 [%]	0.423	SAPS II	0.373
INR	0.467	WBC [10E9/L]	0.373
Diastolic blood pressure [mmHg]	0.478	Bilirubin [mg/dL]	0.414
Lipase [U/L]	0.525	Neutrophils [%]	0.455
Alcoholism	0.532	Sodium+ [mmol/L]	0.480
Alkalosis (pH>7.45)	0.561	Lactate [mmol/L]	0.512

Charlson Comorbidity Index	0.567	SAM [nmol/l]	0.518
FiO2	0.595	Acidosis (pH<7.35)	0.520
Gender	0.646	PCO2 [mmHg]	0.585
Hb [g/dL]	0.683	Cardiovascular diseases	0.595
Eosinophils [%]	0.709	ALT [U/L]	0.704
Sodium+ [mmol/L]	0.763	Alkalosis (pH>7.45)	0.710
PCT [µg/L]	0.772	MTA [nmol/l]	0.757
WBC [10E9/L]	0.780	ScvO2 [%]	0.779
Mechanical ventilation	0.788	PCT [µg/L]	0.825
SAM [nmol/l]	0.834	Age [yrs]	0.854
RASS	0.849	Chloride- [mmol/l]	0.897
Thrombocytes [10E9/L]	0.861	INR	0.912
PCO2 [mmHg]	0.899	Diastolic blood pressure [mmHg]	0.921
Monocytes [%]	0.926	Lipase [U/L]	0.924
Neutrophils [%]	0.946	Thrombocytes [10E9/L]	0.939
MTA [nmol/l]	0.966	Monocytes [%]	0.963
Lymphocytes [%]	0.979	Alcoholism	0.966
		Respiratory diseases	0.986
		Eosinophils [%]	0.987
		FiO2	0.991

*List of non-significant p-values in univariate testing for prediction of progression of disease in descending order; Abbreviations: AST=aspartate aminotransaminase; TISS=therapeutic intervention score scale; ScvO2=central venous oxygen saturation; PO2=partial pressure of oxygen; BEstand=standard base excess; HCO3-stand=standard bicarbonate; ALT=alanine aminotransaminase; SIDa=strong ion difference apparent; SpO2=peripheral capillary oxygen saturation; INR=international normalized ratio; FiO2=fraction of inspired oxygen; Hb=hemoglobin; PCT=procalcitonin; WBC=white blood cell count; SAM=S-adenosylmethionine; RASS=Richmond agitation sedation scale; PCO2=partial pressure of carbon dioxide; MTA=methylthioadenosine.
