# Supplementary material for: S-Adenosylhomocysteine Is a Useful Metabolic Factor in the Early Prediction of Septic Disease Progression and Death in Critically Ill Patients: A Prospective Cohort Study
Source: Int J Mol Sci. 2023 Aug 9;24(16):12600. doi: 10.3390/ijms241612600 (PMC10454796; doi:10.3390/ijms241612600)
Supplement: Supplementary file 1 [file ijms-24-12600-s001.zip › Supplementary Table S3.pdf]

Table S3. Univariate analysis for prediction of death for variables which were tested non-significant.*

	Death
**Variable**	**p-value**
Horovitz Index [mmHg]	0.053
Lactate [mmol/L]	0.066
TISS	0.066
SpO2 [%]	0.071
Respiratory diseases	0.081
Cardiovascular diseases	0.094
Pressor therapy	0.096
Gender	0.116
Respiratory rate [1/min]	0.152
Diastolic blood pressure [mmHg]	0.163
Chloride- [mmol/L]	0.167
PCO2 [mmHg]	0.172
Sodium+ [mmol/L]	0.173
MTA [nmol/l]	0.174
Mean arterial pressure [mmHg]	0.190
Volume balance [mL]	0.190
RASS	0.239
Charlson Comorbidity Index	0.253
SAM [nmol/l]	0.270
FiO2	0.271
Eosinophils [%]	0.333
WBC [10E9/L]	0.347
Systolic blood pressure [mmHg]	0.375
ScvO2 [%]	0.391
Diabetes	0.411
Ionized Calcium++ [mmol/L]	0.419
Shock index	0.431
Alkalosis (pH>7.45)	0.472
CRP [mg/L]	0.494
PCT [µg/L]	0.497
Mechanical ventilation (y)	0.505
Basophils [%]	0.525
INR	0.545
Glucose [mg/dL]	0.611
SIDa [mmol/L]	0.628
Bilirubin [mg/dL]	0.670
PO2 [mmHg]	0.762
Heart rate [1/min]	0.829
AST [U/L]	0.830
ALT [U/L]	0.835
Thrombocytes [10E9/L]	0.852
Neutrophils [%]	0.874

Lipase [U/L]	0.880
Hb [g/dL]	0.899
Monocytes [%]	0.972
Alcoholism	0.973
Temperature [°C]	0.993
Lymphocytes [%]	1.000

*List of non-significant p-values in univariate testing for mortality prediction in descending order; Abbreviations: TISS=therapeutic intervention score scale; SpO2=peripheral capillary oxygen saturation; PCO2=partial pressure of carbon dioxide; MTA=methylthioadenosine; RASS=Richmond agitation sedation scale; SAM=S-adenosylmethionine; FiO2=fraction of inspired oxygen; WBC=white blood cell count; ScvO2=central venous oxygen saturation; CRP=c-reactive protein; PCT=procalcitonin; INR=international normalized ratio; SIDa=strong ion difference apparent; PO2=partial pressure of oxygen; AST=aspartate aminotransaminase; ALT=alanine aminotransaminase; Hb=hemoglobin.
