# Supplementary material for: S-Adenosylhomocysteine Is a Useful Metabolic Factor in the Early Prediction of Septic Disease Progression and Death in Critically Ill Patients: A Prospective Cohort Study
Source: Int J Mol Sci. 2023 Aug 9;24(16):12600. doi: 10.3390/ijms241612600 (PMC10454796; doi:10.3390/ijms241612600)
Supplement: Supplementary file 1 [file ijms-24-12600-s001.zip › Supplementary Table S4.pdf]

Table S4. Pre-analysis: Association of patients with versus without final study inclusion criteria with subsequent onset of severe sepsis and/or septic shock.

	**Development of severe sepsis and/or septic shock**	**No development of severe sepsis and/or septic shock**	**Marginal Row Totals**
**SIRS or sepsis**	19	58	77
**neither SIRS nor sepsis**	6	98	104
**marginal column totals**	25	156	181

*From May 2016 to December 2016 a total of 413 patients were rated daily by a senior intensivist according to sepsis-1/2 defined criteria to suffer from neither SIRS nor sepsis, SIRS, sepsis, severe sepsis, or septic shock. A chi-square test of independence was performed to examine the relation between the presence of SIRS or sepsis for two consecutive days and 24 hours, respectively upon ICU admission (=final study inclusion criteria) and subsequent development of severe sepsis and/or septic shock within the next five days. The relation was significant, $X^2 = 13.28$ $p<0.001$. Following admission to the ICU, critically ill patients diagnosed with SIRS or sepsis for two consecutive days or 24 hours, respectively were more likely to develop severe sepsis and/or septic shock within the following five days than those without. In this pre-analysis 232 patients of the daily rated 413 patients were not included for the following reasons: duration of SIRS less than 48 hours, stay on ICU less than 5 days, initial presentation with severe sepsis or septic shock, no information available from the questionnaire or lack of the questionnaire.
