# Supplementary material for: S-Adenosylhomocysteine Is a Useful Metabolic Factor in the Early Prediction of Septic Disease Progression and Death in Critically Ill Patients: A Prospective Cohort Study
Source: Int J Mol Sci. 2023 Aug 9;24(16):12600. doi: 10.3390/ijms241612600 (PMC10454796; doi:10.3390/ijms241612600)
Supplement: Supplementary file 1 [file ijms-24-12600-s001.zip › Supplementary Table S5.pdf]

Table S5: Diagnostic sepsis 1/2-definition criteria for sepsis, severe sepsis and septic shock*.

**SIRS (≥ 2 of 4 criteria fulfilled)**
Temperature ≥ 38.0°C or < 35.5°C
Heart rate > 90/min or catecholamine requirement ≥ 4h (dose independent)
Respiratory rate > 20/min or p_aCO_2 < 32mmHg or ventilation/NIV therapy
leukocytosis > 12.000/µl or leukopenia < 4.000/µl or > 10% immature neutrophils in differentia blood count
**Sepsis (SIRS + infection)**
confirmed infection
microbiologically confirmed infection
urgent suspicion of infection
**Severe sepsis (Sepsis + organ dysfunction ≥ 2 criteria fulfilled)**
Sepsis-induced hypotension (SBP < 90mmHg or MAP <70mmHg or drop in SBP ≥ 40mmHg from baseline)**
Lactate ≥ 2 mmol/L**
Urine output < 0,5 mL/kg/h for 6-12h (=KDIGO stadium I)**
new onset creatinine increase of 1,5-1,9 times from baseline (=KDIGO Stadium I)**
acute respiratory failure paO_2/FiO_2 < 250 in the absence of pneumonia
acute respiratory failure paO_2/FiO_2 < 200 in presence of pneumonia
new increase of bilirubin > 2mg/dL
new onset of thrombocytopenia < 100.000 µL or platelet transfusion
new onset of coagulopathy (INR > 1,5)
septic encephalopathy (confusion, agitation)
metabolic acidosis (pH < 7,35 und BE < - 4mmol/L)
septic cardiomyopathy (HI < 3,0 L/min/m^2 oder dobutamine-/levosimendan-/milrinone therapy (dose independent))
**Septic shock**
Sepsis-induced hypotension with norepinephrine or epinephrine requirement of ≥ 0,1 µg/kg/min**

*modified from Surviving Sepsis Campaign 2012 ([39, there Table 2) and 2001 International Sepsis Definitions Conference ([40], there Table 1); The applied criteria have been validated previously [41].

**under adequate volume therapy

39. Dellinger, R. P.; Levy, M. M.; Rhodes, A.; Annane, D.; Gerlach, H.; Opal, S. M.; Sevransky, J. E.; Sprung, C. L.; Douglas, I. S.; Jaeschke, R. Surviving Sepsis Campaign: international guidelines for management of severe sepsis and septic shock, 2012. Intensive Care Med **2013**, *39(2)*, 165-228. doi: 10.1007/s00134-012-2769-8.
40. Levy, M. M.; Fink, M. P.; Marshall, J. C.; Abraham, E.; Angus, D.; Cook, D.; Cohen, J.; Opal, S. M.; Vincent, J.-L.; Ramsay, G. 2001 sccm/esicm/accp/ats/sis international sepsis definitions conference. Intensive Care Med **2003**, *29(4)*, 530-538. doi: 10.1007/s00134-003-1662-x.
41. Centner, F. S.; Oster, M. E.; Dally, F. J.; Sauter-Servaes, J.; Pelzer, T.; Schoettler, J. J.; Hahn, B.; Fairley, A. M.; Abdulazim, A.; Hackenberg, K. A. M.; Groden, C.; Etminan, N.; Krebs, J.; Thiel, M.; Wenz, H.; Maros, M. E. Comparative Analyses of the Impact of Different Criteria for Sepsis Diagnosis on Outcome in Patients with Spontaneous Subarachnoid Hemorrhage. J Clin Med **2022**, *11(13)*, 3873. doi: 10.3390/jcm11133873
